# Determinants of impaired growth among hospitalized children – a case-control study

**DOI:** 10.1590/S1516-31802004000300008

**Published:** 2004-05-06

**Authors:** Marilia de Carvalho Lima, Maria Eugênia Farias Almeida Motta, Eliane Cavalcanti Santos, Gisélia Alves Pontes da Silva

**Keywords:** Protein-energy malnutrition, Socioeconomic factors, Low birth weight infant, Healthcare sector, Malnutrition, Desnutrição protéicoenergética, Fatores socioeconômicos, Baixo peso ao nascer, Setor de assistência à saúde, Desnutrição, Morbidade infantil

## Abstract

**CONTEXT:**

Protein energy malnutrition constitutes a public health problem, especially in less affluent countries. The identification of amenable predictive risk factors is of major importance for policy makers to plan interventions to reduce infant malnutrition.

**OBJECTIVE:**

To identify risk factors for protein energy malnutrition among hospitalized low-income children aged 6 to 24 months.

**TYPE OF STUDY:**

Case-control study.

**SETTING:**

Two public hospitals in Recife, Brazil.

**PARTICIPANTS:**

The cases were 124 infants with length-for-age below the 10th percentile of the National Center for Health Statistics curve and the controls were 241 infants with length-for-age equal to or above the 10th percentile who were recruited in the same infirmary.

**METHODS:**

Cases and controls were compared in relation to a variety of sociodemographic, environmental and reproductive factors, and their healthcare, previous feeding practice and morbidity. Logistic regression analysis was used to investigate the net effect of risk factors on infant malnutrition, after adjusting for potential confounding variables.

**RESULTS:**

The mother's age, possession of a TV set, type of water supply, family size and location of the home were significantly associated with child malnutrition in the bivariate analysis. However, these associations lost their significance after adjusting for other explanatory variables in the hierarchical logistic regression analysis. This analysis showed that low birth weight contributed the largest risk for impaired growth. Increased risks of infant malnutrition were also significantly associated with households that had no toilet facilities or refrigerator, high parity for the mother, no breastfeeding of the infant, inadequate vaccination coverage and previous hospitalization for diarrhea and pneumonia.

**DISCUSSION:**

The literature shows that chronic malnutrition, as assessed by low length-for-age indexes, is often related to low income. However, this was not the case in this study, in which other variables had greater impact on child growth.

**CONCLUSIONS:**

In view of the multiple causes of malnutrition, the interrelationship among its determinants should be taken into account when adopting strategies for its reduction and prevention.

## RESUMO

**CONTEXTO:**

Desnutrição energético-protéica constitui um problema de saúde pública especialmente em países em desenvolvimento. A identificação de fatores de risco passíveis de controle é de grande importância para os sanitaristas planejarem intervenções a fim de reduzirem a desnutrição na infância.

**OBJETIVOS:**

Identificar fatores de risco para desnutrição energético-protéica em crianças hospitalizadas e de baixa renda com 6 a 24 meses de idade.

**TIPO DE ESTUDO:**

Estudo do tipo casocontrole.

**LOCAL DO ESTUDO:**

Instituto Materno Infantil de Pernambuco e Hospital Barão de Lucena, dois hospitais públicos do Recife, nordeste do Brasil.

**PARTICIPANTES:**

Casos foram 124 crianças com comprimento para a idade abaixo do 10° percentil para a curva do National Center for Health Statistics e controles foram 241 crianças com comprimento para a idade igual ou acima do 10° percentil e recrutadas na mesma enfermaria.

**MÉTODOS:**

Casos e controles foram comparados em relação a uma série de fatores sociodemográficos, ambientais, reprodutivos, assistência à saúde, prática alimentar e morbidades prévias. Análise de regressão logística foi utilizada a fim de investigar o efeito independente dos fatores de risco para desnutrição infantil, após ajuste para potenciais fatores de confusão. A estratégia analítica usada foi o modelo hierárquico que consiste na entrada das variáveis explanatórias no modelo em uma ordem previamente estabelecida pelo pesquisador, baseada em um modelo conceitual que descreve as relações lógicas e teóricas entre os fatores de risco.

**RESULTADOS:**

A idade materna, posse de aparelho de TV, tipo de abastecimento de água, tamanho da família e local de moradia apresentaram associação significante com a desnutrição; no entanto, essas associações perderam a significância depois de serem controladas por outras variáveis explanatórias na análise de regressão logística hierarquizada. Esta análise mostrou que o baixo peso ao nascer contribuiu com o maior risco para desnutrição na população estudada (odds ratio = 6,04; p = 0,005). Maiores riscos para desnutrição também foram significantemente associados com habitações sem sanitário e sem geladeira, multiparidade, nunca ter sido amamentado, vacinação desatualizada e hospitalização prévia por diarréia e pneumonia.

**DISCUSSÃO:**

A literatura mostra que a desnutrição crônica, apontada por índices de altura-para-idade baixos, está freqüentemente associada a baixa renda. Isto não foi verificado neste estudo, em que outras variáveis, tais como peso baixo ao nascer e acesso a sanitários e refrigeradores, além da paridade da mãe, amamentação, vacinação e história de diarréia e pneumonia, tiveram maior impacto sobre o crescimento das crianças.

**CONCLUSÕES:**

Em vista da multicausalidade da desnutrição, as interrelações entre seus determinantes devem ser levadas em consideração ao se adotar estratégias para a sua redução e prevenção.

## INTRODUCTION

Protein energy malnutrition is a major public health problem in childhood affecting a great number of children, especially in developing countries. Its determinants are of biological origin (low birth weight, early weaning and inadequate healthcare) and social origin (unfavorable socioeconomic and environmental conditions). These factors are interrelated, with each one contributing to the occurrence and persistence of the other factors, and they act directly (biologically) or indirectly (socially) on the nutritional status.^1,2^

The relationship of socioeconomic and environmental factors with the nutritional status of under-five children has been registered in many studies.^3,4^ In developing countries, protein energy malnutrition has been associated with the state of poverty and is an important indicator of the quality of life of a population.^2,5^ The economic conditions of the household establish its purchasing power and indirectly determine the food consumption of its members.^2^ At the same time, simple and low-cost measures such as basic sanitation and immunization prevent the adverse effects of diseases on the nutritional status during childhood.^2^

Adequate weight at birth is an important determinant of normal growth, and low birth weight babies (< 2,500 g) are at increased risk of remaining malnourished during childhood. Thus, reduction of the prevalence of low birth weight in a specific area is a fundamental measure for improving the nutritional status of children.^2,6,7^ Early weaning is another condition frequently associated with malnutrition among children belonging to low-income families.^8^ Breast milk supplies the nutrients for adequate growth and contributes towards reducing the occurrence of infectious diseases during the first months of life.^2^ Early introduction of overdiluted and contaminated formulae makes infants more susceptible to acquiring infectious diseases, especially diarrhea.^2^Repeated episodes of acute diarrhea and other infections, as well as food shortages, contribute towards the commencement or worsening of malnutrition as a result of impaired absorption of nutrients, thereby causing growth to falter.^2,9^ Protein energy malnutrition therefore has a multifactorial origin, with a complex interrelationship among its determinants.

This study had the aim of identifying the determinants of malnutrition among hospitalized infants in the age group from 6 to 24 months.

## METHODS

### Setting

The study was conducted in two hospitals in Recife, the capital of the State of Pernambuco, located in the northeastern region of Brazil. Instituto Materno Infantil de Pernambuco and Hospital Barão de Lucena are the largest public referral hospitals for pediatric attendance in the metropolitan area of the city. Both hospitals belong to the Brazilian National Health System (Sistema Único de Saúde — SUS) and mostly serve low-income families. According to official figures, over half of the population of Recife lives in shanty towns as a result of the rapid urbanization that has taken place in most Latin American cities. At the time of the study, the infant mortality rate was 35 per 1,000 live births and the prevalence of children under five years old with z-scores for weight-for-age and length-for-age of less than −2 was 3.5% and 9.4%, respectively, for the Metropolitan Region of Recife.^10^

### Type of study and sample size

The design adopted was a case-control study. A total of 124 cases and 241 controls, in the age group from 6 to 24 months, were recruited between January and October 1997. This sample gave an 80% power for detecting an odds ratio (OR) of ³ 2.1 with significance at the level of 5%, for a prevalence of exposure among controls varying from 24 to 45%.

### Selection of cases and controls

The group of cases was constituted by infants with length-for-age below the 10th percentile of the reference curve of the American population drawn up by the National Center for Health Statistics (NCHS).^11^ The infants forming the controls had length-forage equal to or above the 10th percentile of the NCHS classification. The cases and controls were recruited from the same pediatric wards of both hospitals, in the proportion of one case for two controls. In order to avoid imbalance in the age distribution, the controls were matched for age in relation to the group of cases, with a maximum difference of ± 3 months. The exclusion criteria applied to both groups were the presence of underlying conditions that lead to the faltering of growth, such as chronic diseases, congenital malformations and chromosomal anomalies.

### Data collection

Assessment of risk factors for child malnutrition was accomplished through inquiry among the mothers using a standardized precoded questionnaire, following recruitment of their children. The questionnaire was pre-tested to ensure that the questions were comprehensible to these mothers.

The anthropometric measurements were practiced in advance of the survey, and the equipment was also checked. The anthropo-metric measurements were assessed according to standard techniques defined by Gibson.^12^

The children were weighed without clothes on a calibrated baby scale (Filizola, São Paulo, Brazil) with a capacity of 16 kg and precision of 10 g. Length was measured using a portable infantometer (Pedobaby), to the nearest 0.1 cm.

Ethical approval for the study was obtained from the Research Ethics Committee of the Center for Health Sciences, Universidade Federal de Pernambuco.

### Data recording and analysis

Data were coded regularly and checked for consistency, accuracy and completeness. Double data entry was conducted on an IBM-compatible microcomputer, using the EpiInfo version 6.0 software (CDC, Atlanta)^13^ to verify cross-checking. Statistical analysis was undertaken using the Statistical Package for the Social Sciences, version 8.0 for Windows (SPSS Inc., Chicago, Illinois).^14^ Bivariate analysis was conducted between the dependent variable and each one of the potential determinants of malnutrition: household socioeconomic indicators, birth weight, total duration of breastfeeding, immunization coverage, previous hospitalization and mother's age and parity. The baseline category for estimation of the crude and adjusted OR was the category with the smallest risk for child malnutrition. The chi-squared test was used to assess the strength of the association, and statistical significance was taken as p ≤ 0.05.

Logistic regression analysis was used to investigate the net effect of risk factors on infant malnutrition, after adjusting for potential confounding variables. The analytical strategy adopted was the hierarchical approach, which consists of entering the explanatory variables in the model one at a time in an order previously specified by the researcher, on the basis of a model describing the logical or theoretical relationship between the risk factors.

By adopting this approach, five regression levels were developed. Firstly, the socioeconomic and demographic variables (per capita family income, mother's education, possession of radio, refrigerator and television, family size, location of the home and cohabitation with infant's father) were placed in the highest hierarchical level, since these may directly or indirectly determine all the factors studied. They were then regressed against length-for-age. The second hierarchical level consisted of the environmental factors (construction material for the house walls, type of toilet and water supply), which are partly determined by socioeconomic conditions. Following these, the reproductive factors (mother's age and parity) were included in the third level of the model. The fourth level was constructed from the preceding levels, with the inclusion of birth weight. Finally, the fifth level of the model brought in the variables relating to childcare and morbidity history (duration of breastfeeding, vaccination coverage and previous hospitalization for diarrhea and pneumonia).

Variables that continued to be ‘significant’ at the level of 20% were kept in the model and participated in the adjustment of the next level. Once selected in a given level, they remained in the subsequent models, even if their significance was lost through the inclusion of variables placed in an inferior hierarchical level.

## RESULTS

The total sample consisted of 365 infants, and length-for-age below the 10th percentile was found in 124 infants (34%). Table 1 shows that the families were largely poor and half of them (53%) had incomes below the poverty line (half of the per capita monthly minimum wage, equivalent to US$ 50 in 1997 and most of them (63%) were living in households with 5 people or more, with limited sanitation. Around 62% of the women had never been to school or had less than five years of schooling, and 17% were adolescents. Low birth weight was found in 7.5% of the sample.

**Table 1:** Selected characteristics of 365 infants in Recife region

Variables	Index length for age				Total	
	< P_10_		≤ P_10_			
	n	%	n	%	n	%
Per-capita family income						
≤ 0.50 minimum wage	94	75.8	100	41.5	194	53.2
> 0.50 minimum wage	15	12.1	98	40.7	113	30.9
Unknown	15	12.1	43	17.8	58	15.9
Water supply						
Inside the house	52	41.9	169	70.1	221	60.5
Outside the house	27	21.8	31	12.9	58	15.9
Others	45	36.3	41	17.0	86	23.6
Type of toilet						
Flush toilet	36	29.0	162	67.2	198	54.2
Pit latrine	52	42.0	65	27.0	117	32.1
None	36	29.0	14	5.8	50	13.7
Presence of refrigerator at home	27	21.8	146	60.6	173	47.4
Presence of television at home	70	56.5	187	77.6	257	70.4
Number of persons in the household						
2-4	34	27.4	100	41.5	134	36.7
5-7	57	46.0	104	43.2	161	44.1
≥ 8	33	26.6	37	15.3	70	19.2
Mother's schooling (years)						
0 – 4	102	82.3	123	51.0	225	61.6
≥ 5	22	17.7	118	49.0	140	38.4
Mother's age (years)*						
13 – 19	19	15.4	43	17.8	62	17.1
20 – 29	67	54.5	153	63.5	220	60.4
≥ 30	37	30.1	45	18.7	82	22.5
Birth weight**						
1,500-2,499	16	15.1	9	4.0	25	7.5
2,500-2,999	25	23.6	45	19.8	70	21.0
3,000-3,499	36	34.0	102	44.9	138	41.5
≥ 3,500	29	27.3	71	31.3	100	30.0

*P = percentile;*

* *1 case without information;*

** *32 cases without information.*

The mother's age and cohabitation with the father, possession of a TV set, family size, water supply and location of the home were excluded from the regression analysis, because they did not attain the statistical “significance” required for them to remain in the model (Table 2).

**Table 2:** Nutritional status of 365 children in Recife according to socioeconomic, demographic and environmental indicators

Variables	Index length for age		
	< P_10_	≤ P_10_	Raw OR (95% CI)
Mother's age (years)*			
20-29	67	153	1.00
13-19	19	43	1.01 (0.55-1.86)
≥ 30	37	45	1.93 (1.15-3.24)†
Cohabitation with father			
Yes	94	201	1.00
No	30	40	1.60 (0.94-2.73)‡
Presence of television at home			
Yes	70	187	1.00
No	54	54	2.67 (1.68-4.26)§
Number of persons in the household			
2-4	34	100	1.00
5-7	57	104	1.61 (0.97-2.67)
≥ 8	33	37	2.62 (1.43-4.83)^II^
Water supply			
Inside the house	52	169	1.00
Outside the house	27	31	2.83 (1.55-5.17)§
Others	45	41	3.57 (2.11-6.03)§
Location of the home ¶			
Metropolitan region of Recife	62	148	1.00
Urban area (other cities)	29	68	0.99 (0.59-1.67)
Rural area	33	23	3.43 (1.86-6.30)§

*P = percentile; OR = odds ratio; CI = confidence interval;*

* *one case without information;*

† *p < 0.05;*

‡ *p < 0.10;*

§ *p < 0.001;*

II *p < 0.01;*

¶ *two cases without information.*

The variables that continued to show significance in the logistic regression analysis for explaining protein energy malnutrition were the type of toilet, possession of refrigerator, parity, birth weight, duration of breastfeeding, vacci-nation coverage and previous hospitalizations for diarrhea and pneumonia. The largest risk for malnutrition was found in relation to children of low birth weight: a risk that was around six times higher than for children with birth weight of 3,500 g or more (Table 3).

**Table 3:** Hierarchical logistic regression analysis of risk factors for malnutrition among 365 hospitalized infants

Variables	Index length for age			
	< P_10_	≥ P_10_	Raw OR (95% CI)	Adjusted OR (95% CI)
*Per capita* family income				
> 0.50 minimum wages	15	98	1.00	1.00
Unknown	15	43	2.28 (1.02-5.07)*	2.06 (0.74-5.72)
≤ 0.50 minimum wages	94	100	6.14 (3.33-11.31)†	1.85 (0.80-4.27)
Construction material for house walls				
Brick and cement	67	201	1.00	1.00
Others	57	40	4.27 (2.62-6.98)†	1.91 (0.88-4.15)
Type of toilet				
Flush toilet	36	162	1.00	1.00
Pit latrine	52	65	3.60 (2.15-6.01)†	0.89 (0.40-1.98)
None	36	14	11.57 (5.66-23.66)†	4.07 (1.30-12.71)*
Presence of radio at home				
Yes	88	215	1.00	1.00
No	36	26	3.38 (1.93-5.93) †	1.54 (0.65-3.66)
Presence of refrigerator at home				
Yes	27	146	1.00	1.00
No	97	95	5.52 (3.35-9.09)†	2.25 (1.02-4.95)*
Mother's schooling (years)				
≥ 5	22	118	1.00	1.00
0 – 4	102	123	4.45 (2.63-7.52)†	1.04 (0.48-2.26)
Parity				
1	13	72	1.00	1.00
2 - 4	66	143	2.56 (1.32-4.94)§	1.76 (0.72-4.32)
≥ 5	45	26	9.58 (4.47-20.55) †*	4.54 (1.48-13.97)§
Birth weight (g)‡				
≥ 3,500	29	71	1.00	1.00
3,000-3,499	36	102	0.86 (0.49-1.54)	0.62 (0.28-1.36)
2,500-2,999	25	45	1.36 (0.71-2.61)	1.54 (0.63-3.80)
1,500-2,499	16	9	4.35 (1.73-10.96)§	6.04 (1.73-21.08)§
Duration of breastfeeding (months)				
≥ 7	16	79	1.00	1.00
4-6	33	76	2.14 (1.09-4.21)*	1.18 (0.47-2.97)
1-3	48	56	4.23 (2.18-8.20)†	2.13 (0.85-5.33)
None	27	30	4.44 (2.10-9.38)†	3.30 (1.16-9.40)*
Vaccination schedule for age II				
Completed	22	129	1.00	1.00
Uncompleted	92	106	5.09 (2.99-8.65)†	3.02 (1.49-6.10)§
Previous hospitalization for pneumonia				
No	96	212	1.00	1.00
Yes	28	29	2.13 (1.20-3.78)§	2.43 (1.06-5.57)*
Previous hospitalization for diarrhea				
No	78	216	1.00	1.00
Yes	46	25	5.10 (2.94-8.85)†	2.99 (1.33-6.71)§

*P = percentile; OR = odds ratio; CI = confidence interval;*

* *p < 0.05;*

† *p < 0.001;*

‡ *32 cases without information;*

§ *p < 0.01;*

II *16 cases without information.*

## DISCUSSION

The influence of socioeconomic and environmental conditions on child nutrition has been widely studied and some indicators like the mother's education level, family size and household conditions have been identified as risk factors.^3,4^ Child nutrition assessed through length-for-age has been associated with socioeconomic conditions and low birth weight.^1^ The results of the present study confirm the multiple causes of protein energy malnutrition that are described in the literature.^1,2^

Most of the factors were poverty-related and we had expected that per capita family income below the poverty line would be a significant determinant of child protein energy malnutrition, but this proved not to be the case after adjusting for the other variables. Reported income, however, is known to be generally unreliable, and intermittent casual income was not surveyed in this study. The families in the “unknown” income category (58 cases) tended to have very few possessions and their non-reporting of income may reflect embarrassment about their impoverished circumstances.

The mother's education level can directly influence child health through the adoption of preventive care (breastfeeding, hygiene and immunization) and curative care (appropriate treatment of diseases) or indirectly influence it through better employment and opportunities and income.^4,15,16^ Children whose mothers had less than five years of schooling had an unadjusted risk of developing protein energy malnutrition that was 4.4 times higher than for those whose mothers had studied for five years or more (p < 0.001). This has also been found in other studies, as well as in two large national household surveys: Pesquisa Nacional sobre Saúde e Nutrição (PNSN) and Pesquisa Nacional sobre Demografia e Saúde (PNDS), conducted in 1989 and 1996, respectively.^1,4,17-22^ However, in our study the mother's number of years of schooling lost its significance after adjusting for environmental variables. The environmental conditions are probably more important than the mother's education level in fostering adequate child health.^15^

The possession of a refrigerator at home and the type of toilet were socioeconomic indicators that remained significant in the regression analysis (p < 0.05 for each variable). Possession of a refrigerator also indicates that the power to purchase some household appliances can have a benefit in terms of child nutrition, since appropriate storage of foods prevents its waste and contamination. Children living in households without a latrine were more undernourished than those with a flush toilet at home. This finding has also been observed in other studies.^4,23^

Children from rural areas were significantly more undernourished than those from the metropolitan area of Recife. However, this significant association was lost after adjusting for other socioeconomic and environmental variables. The association between nutritional status and location of the home can have the possible confounding factor of environmental conditions, since only a few rural households have facilities like piped water and flush toilets.^4^ It is possible that appropriate environmental conditions, which are essential for preventing infectious diseases, are more important than the location of the home for ensuring satisfactory growth.^1,15,19,24^

The risk of growth retardation is higher for infants of mothers with high parity.^4,22,24^ It is well known that the time dedicated to childcare in the case of many siblings impairs the quality of the mother's attention. Breastfeeding of the younger infants can be harmed, while the care of the older ones becomes neglected, thereby contributing to deficient feeding and consequent malnutrition.^1,4,24,25^ According to Vaahtera et al.,^25^ family planning may improve adherence to exclusive breastfeeding and feeding recommendations at the time of weaning. In the present study, children whose mothers had five children or more had a risk of protein energy malnutrition that was five times greater than for those with one child only (p < 0.01).

Low birth weight is associated with growth deficit that settles down after the post-natal period.^1,2,8^ These children are more vulnerable to diseases, frequently have a history of breastfeeding failure and are at a disadvantage in relation to growth, when compared with those of appropriate weight at birth.^6,8,26^

The present study showed a significant association between low birth weight and protein energy malnutrition, even after adjusting for other variables (p < 0.01). Other studies have found the same results.^1,8,26,27^

In the first months of life, the amount and quality of breast milk are appropriate for normal growth, as well as for catch-up growth after episodes of diseases.^28^ Early weaning or absence of breastfeeding is an important risk factor for protein energy malnutrition.^18,29,30^

During weaning, when the protection provided by breast milk disappears, there is a reduction in the consumption of foods and an increase in the frequency of diarrhea.^31^ In the present study, children that had no breast-feeding were significantly more undernourished than those who were breastfed for more than six months, after controlling for confounding variables (p < 0.05). It is possible that the food intake of such children lacked the fundamental nutritional elements for fostering satisfactory growth, thereby facilitating the onset of protein energy malnutrition.

Community vaccination programs can substantially contribute to health in early childhood.^23^ Immunization is a public health measure that has an essential impact on the nutritional status of children, because it avoids the negative effects of serious diseases like measles and whooping cough.^30^ It was observed in the present study that children with uncompleted immunization had a risk of developing protein energy malnutrition that was around three times higher than for those with completed immunization, even after adjusting for other risk factors (p < 0.01).

One of the main immediate causes of failure to thrive after the fourth or fifth month of life are the infectious diseases, especially diarrhea and acute respiratory infection.^31^ The negative effect of diarrhea on nutrition is caused by reduced food intake due to anorexia, malabsorption and metabolic changes.^2^ Some authors have documented an association between hospitalization for diarrhea and nutritional deficit.^4,32,33^ Hospitalization for diarrhea (p < 0.01) and pneumonia (p < 0.05) contributed significantly, with a risk for the onset of protein energy malnutrition that was three times greater than for children that were not hospitalized because of such morbidities. These findings confirm the deleterious effect of such diseases on the nutritional status and point out the need to reduce child morbidity so as to prevent the impairment of growth.^33^

## CONCLUSIONS

The quality of the environment is a determinant of health and nutrition and thus should be considered in the evaluation of the nutritional status. For individual prevention of nutritional changes in infancy, not only the economic, social and demographic conditions surrounding the mother and the child should be observed, but also their healthcare and risk of morbidities. Prenatal attendance needs to be a priority in public health, since low birth weight was detected as the factor with the greatest contribution to the risk of infant malnutrition in the multivariate analysis. The study demonstrated that several factors were responsible for protein energy malnutrition, and the influence of low birth weight and unfavorable socioeconomic and environmental conditions were prominent among such factors. The social aspects should be strongly considered when planning measures to improve infant health and nutrition.

## References

[1] Olinto MTA, Victora CG, Barros FC, Tomasi E (1993). Determinantes da desnutriçao infantil em uma populaçao de baixa renda: um modelo de análise hierarquizado. [Determinants of malnutrition in a low-income population: hierarchical analytical model]. Cad Saúde Pública.

[2] Batista M, Rissin A (1993). Deficiências nutricionais: ações específicas do setor saúde para o seu controle. [Nutritional deficiencies: specific control measures by the health sector]. Cad Saúde Pública.

[3] Batrouni L, Pérez-Gil SE, Rivera J, González de Cosío T (1985). Differentiation of the nutritional status of preschool children, according to socioeconomic levels, in a marginal zone. [Differentiation of the nutritional status of preschool children, according to socioeconomic levels, in a marginal zone]. Arch Latinoam Nutr.

[4] Victora CG, Vaughan JP, Kirkwood BR, Martines JC, Barcelos LB (1986). Risk factors for malnutrition in Brazilian children: the role of social and environmental variables. Bull World Health Organ.

[5] Batista M, Silva DO, Sousa H (1992). Desnutriçao em crianças de áreas faveladas: Manguinhos, Rio de Janeiro. [Undernutrition in children from areas of urban slums: Manguinhos, Rio de Janeiro]. Cad Saúde Pública.

[6] Victora CG, Barros FC, Vaughan JP, Teixeira AM (1987). Birthweight and infant mortality: a longitudinal study of 5914 Brazilian children. Int J Epidemiol.

[7] Leone C, Mascaretti LAS, Primo E, Yamamoto TS, Freschi SA (1992). Peso de nascimento e características médico-sociais. [Birth weight and social-medical characteristics]. J Pediatr.

[8] Nóbrega FJ, Vítolo MR, Queiroz SS, Magrini JE (1994). Crianças desnutridas internadas: relação com variáveis maternas. [Mal-nourished children hospitalized: relations with maternal variables]. Rev Paul Pediatr.

[9] Guerrant RL, Hughes JM, Lima NL, Crane J (1990). Diarrhea in developed and developing countries: magnitude, special settings, and etiologies. Rev Infect Dis.

[10] INAN (Instituto Nacional de Alimentação e Nutrição), IMIP (Instituto Materno Infantil de Pernambuco), DN/UFPE (Departamento de Nutrição da Universidade Federal de Pernambuco), SES/PE (Secretaria de Saúde do Estado de Pernambuco) (1998). II Pesquisa Estadual de Saúde e Nutrição: saúde, nutrição, alimentação e condições socioeconômicas no Estado de Pernambuco.

[11] World Health Organization (1983). Measuring change in nutritional status: guidelines for assessing the nutritional impact of supplementary feeding programmes for vulnerable groups.

[12] Gibson RS (1990). Principles of nutritional assessment.

[13] CDC (Centers for Disease Control and Prevention) (1994). Epi Info, version 6.04. Um sistema de processamento de texto, banco de dados e estatística para Saúde Pública em microcomputadores IBM-compatíveis.

[14] SPSS Inc (1998). Statistical Package for the Social Sciences, version 8.0 for Windows.

[15] Monteiro CA, Pino Zuñiga HP, Benício MHDA, Szarfarc SC (1986). Estudo das condiçöes de saúde das crianças do município de Säo Paulo, SP (Brasil), 1984-1985: I. Aspectos metodológicos, caracteristicas sócio-econômicas e ambiente físico. [Health conditions of children of the municipality of São Paulo, SP (Brazil), 1984-1985: I. Methodological aspects, socioeconomic characteristics and physical environment]. Rev Saúde Pública.

[16] Huttly SR, Victora CG, Barros FC, Teixeira AM, Vaughan JP (1991). The timing of nutritional status determination: implications for interventions and growth monitoring. Eur J Clin Nutr.

[17] Delpeuch F, Traissac P, Martin-Prével Y, Massamba JP, Maire B (2000). Economic crisis and malnutrition: socioeconomic determinants of anthropometric status of preschool children and their mothers in an African urban area. Public Health Nutr.

[18] Iqbal Hossain M, Yasmin R, Kabir I (1999). Nutritional and immunisation status, weaning practices and socio-economic conditions of under five children in three villages of Bangladesh. Indian J Public Health.

[19] Engstrom EM, Anjos LA (1999). Déficit estatural nas crianças brasileiras: relaçäo com condiçöes sócio-ambientais e estado nutricional materno. [Stunting in Brazilian children: relationship with social-environmental conditions and maternal nutritional status]. Cad Saúde Pública.

[20] Carvalho NM, Giugliani ERJ, Seffrin CF, Hartmann RM (1992). Seguimento de crianças com desnutriçäo moderada ou grave em populaçäo periférica (Brasil). [Follow up of children with moderate or severe malnutrition in an outlying urban population (Brazil)]. Rev Saúde Pública.

[21] INAN (Instituto Nacional de Alimentação e Nutrição) (1990). Perfil de crescimento da população brasileira de 0 a 25 anos. Pesquisa Nacional sobre Saúde e Nutrição (PNSN).

[22] BEMFAM (Sociedade Civil Bem Estar Familiar no Brasil) (1997). Pesquisa Nacional sobre Demografia e Saúde. Programa de Pesquisas de Demografia e Saúde.

[23] Paknawin-Mock J, Jarvis L, Jahari AB, Husaini MA, Pollitt E (2000). Community-level determinants of child growth in an Indonesian tea plantation. Eur J Clin Nutr.

[24] Reichenheim ME, Harpham T (1990). An intra-community profile of nutritional deficiency: a study of children under 5 in a low-income community in Rio de Janeiro (Brazil). [An intra-community profile of nutritional deficiency: a study of under −5 in a low-income community in Rio de Janeiro (Brazil)]. Rev Saúde Pública.

[25] Vaahtera M, Kulmala T, Hietanen A (2001). Breastfeeding and complementary feeding practices in rural Malawi. Acta Paediatr.

[26] Victora CG, Barros FC, Vaughan JP, Margines JC, Beria JU (1987). Birthweight, socioeconomic status and growth of Brazilian infants. Ann Hum Biol.

[27] González GJ, Vega MG (1994). Condiciones sociodemograficas y estado nutricional de niños menores de um año en areas perifericas de Guadalajara, México. [Sociodemographic conditions and nutritional status of children under one year old in areas adjacent to Guadalajara, Mexico]. Rev Saúde Pública.

[28] Lunn PG (2000). The impact of infection and nutrition on gut function and growth in childhood. Proc Nutr Soc.

[29] Nóbrega FJ, Campos AL (1996). Distúrbios nutricionais e fraco vínculo mãe/filho.

[30] Bittencourt SA, Leal MC, Gadelha AMJ, Oliveira MA (1993). Crescimento, diarréia e aleitamento materno: o caso da Vila do João. [Growth, diarrhea and breastfeeding: the case of Vila do João]. Cad Saúde Pública.

[31] Engstron EM, Anjos LA (1996). Relaçäo entre o estado nutricional materno e sobrepeso nas crianças brasileiras. [Relationship between maternal nutritional status and overweight in Brazilian children]. Rev Saúde Pública.

[32] Abate G, Kogi-Makau W, Muroki NM (2000). Health seeking and hygiene behaviors predict nutritional status of pre-school children in a slum area of Addis Ababa, Ethiopia. Ethiop Med J.

[33] Kossmann J, Nestel P, Herrera MG, El-Amin A, Fawzi WW (2000). Undernutrition and childhood infections: a prospective study of childhood infections in relation to growth in the Sudan. Acta Paediatr.

